# Meet the Co-Editor

**DOI:** 10.2174/1570159X2105230320095212

**Published:** 2023-04-12

**Authors:** Filippo Caraci

**Affiliations:** 1 Department of Drug and Health Sciences, University of Catania, Catania, Italy

Prof. Caraci graduated MD in 2001 and he got his PhD degree in Neuropharmacology in 2009 at University of Catania. In March 2021 he was appointed Associate Professor in Pharmacology at University of Catania and Visiting Professor at University of Bordeaux in 2016/2017 as winner of the Visiting Scholar Grant 2016/2017. Prof. Caraci has worked in the field of drug discovery in depression, Alzheimer’s disease and Down Syndrome with the aim to identify new pharmacological targets. Prof. Caraci has been studying the physiology and pharmacology of TGF-ß1 and its role in the pathophysiology of cognitive dysfunction in AD and Down Syndrome.
